# No Critical Peripheral Fatigue Threshold during Intermittent Isometric Time to Task Failure Test with the Knee Extensors

**DOI:** 10.3389/fphys.2016.00627

**Published:** 2016-12-19

**Authors:** Christian Froyd, Fernando G. Beltrami, Guillaume Y. Millet, Timothy D. Noakes

**Affiliations:** ^1^Faculty of Teacher Education and Sport, Sogn og Fjordane University CollegeSogndal, Norway; ^2^Department of Human Biology, University of Cape TownCape Town, South Africa; ^3^Exercise Physiology Lab, Department of Health Sciences and TechnologyETH Zurich, Zürich, Switzerland; ^4^Human Performance Laboratory, Faculty of Kinesiology, University of CalgaryCalgary, AB, Canada

**Keywords:** maximal voluntary contraction, femoral nerve electrical stimulation, neuromuscular activation, neuromuscular fatigue, evoked peak force, knee extension, electromyography, rating of perceived exertion

## Abstract

It has been proposed that group III and IV muscle afferents provide inhibitory feedback from locomotor muscles to the central nervous system, setting an absolute threshold for the development of peripheral fatigue during exercise. The aim of this study was to test the validity of this theory. Thus, we asked whether the level of developed peripheral fatigue would differ when two consecutive exercise trials were completed to task failure. Ten trained sport students performed two exercise trials to task failure on an isometric dynamometer, allowing peripheral fatigue to be assessed 2 s after maximal voluntary contraction (MVC) post task failure. The trials, separated by 8 min, consisted of repeated sets of 10 × 5-s isometric knee extension followed by 5-s rest between contractions. In each set, the first nine contractions were performed at a target force at 60% of the pre-exercise MVC, while the 10th contraction was a MVC. MVC and evoked force responses to supramaximal electrical femoral nerve stimulation on relaxed muscles were assessed during the trials and at task failure. Stimulations at task failure consisted of single stimulus (SS), paired stimuli at 10 Hz (PS10), paired stimuli at 100 Hz (PS100), and 50 stimuli at 100 Hz (tetanus). Time to task failure for the first trial (12.84 ± 5.60 min) was longer (*P* < 0.001) than for the second (5.74 ± 1.77 min). MVC force was significantly lower at task failure for both trials compared with the pre-exercise values (both *P* < 0.001), but there were no differences in MVC at task failure in the first and second trials (*P* = 1.00). However, evoked peak force for SS, PS100, and tetanus were all reduced more at task failure in the second compared to the first trial (*P* = 0.014 for SS, *P* < 0.001 for PS100 and tetanus). These results demonstrate that subjects do not terminate exercise at task failure because they have reached a critical threshold in peripheral fatigue. The present data therefore question the existence of a critical peripheral fatigue threshold during intermittent isometric exercise to task failure with the knee extensors.

## Introduction

Neuromuscular fatigue is often defined as a reduction in maximal voluntary contraction (MVC) force. Both (i) central fatigue, defined as a reduction in the maximal capacity of the central nervous system to maximally recruit motor units to produce force and (ii) peripheral fatigue, defined as the reduction in force originating from sites at or distal to the neuromuscular junction (Gandevia, 2001) contribute to neuromuscular fatigue. Peripheral fatigue is commonly measured as a reduction in evoked force responses to electrical or magnetic supramaximal stimulations delivered to the motor nerve to relaxed muscles (Verges et al., 2009; Millet et al., 2011).

It has been proposed that peripheral fatigue is the critical event at task failure (Amann et al., 2006; Amann and Dempsey, 2008) and that group III and IV muscle afferents provide inhibitory feedback from locomotor muscles to the central nervous system (Taylor and Gandevia, 2008), influencing the regulation of central motor drive during fatiguing exercise, and thus playing a key role in determining the moment of exhaustion (Taylor and Gandevia, 2008; Amann, 2012). It has been further proposed that a reduction in central motor drive i.e., a reduction in voluntary descending drive from the primary motor cortex usually indirectly measured via electromyography (EMG) (Amann et al., 2013), constrains the development of peripheral fatigue to a certain “critical” threshold associated with a given level of intramuscular metabolic perturbation (Amann et al., 2006). According to this model, humans may not ever exceed a critical level of peripheral fatigue, leading to the proposal of a critical peripheral fatigue threshold (Amann et al., 2006; Amann and Dempsey, 2008). As a result, when the critical peripheral fatigue threshold is approached, feedback from group III and IV muscle afferents reduces central motor drive and thus exercise intensity during self-paced exercise (Amann and Dempsey, 2008), or triggers task failure during constant load exercise (Amann et al., 2011).

In support of a critical peripheral fatigue threshold, similar levels of peripheral fatigue have been reported after constant-load endurance exercise with different degrees of arterial oxygen content (Amann et al., 2006), after intermittent isometric knee extension to task failure at different intensities (Burnley et al., 2012), after self-paced endurance exercise whether or not subjects were pre-fatigued before exercise (Amann and Dempsey, 2008), and after all-out cycling sprints whether or not subjects were pre-fatigued by electrical stimulation (Hureau et al., 2014). Support for a critical peripheral fatigue threshold is provided by studies showing greater levels of peripheral fatigue at the end of exercise following selective blockade of sensory afferents with intrathecal fentanyl injection compared to saline (Amann et al., 2009, 2011; Blain et al., 2016).

However, a critical peripheral fatigue threshold is not a universal finding, leading some authors to question the importance of peripheral fatigue in regulating exercise performance (Marcora and Staiano, 2010; Christian et al., 2014; Froyd et al., 2016; Neyroud et al., 2016). But these criticisms of this theory have been dismissed on the basis that some studies employed designs in which the interventions produced lower levels of peripheral fatigue than did the control conditions (Johnson et al., 2015). It has been argued (Broxterman et al., 2015) that to disprove the existence of a critical peripheral fatigue threshold, an experimental manipulation must cause the subjects to surpass the threshold, that is, by achieving higher levels of peripheral fatigue in the intervention condition. If inhibitory feedback from group III and IV muscle afferents constrains the extent to which peripheral fatigue develops during endurance exercise (Amann, 2011, 2012), it follows that trials of similar intensity, but different pre-fatiguing conditions will be of different durations, but should finish at similar levels of peripheral fatigue.

Therefore, the aim of this study was to test the validity of the critical peripheral fatigue threshold model during exercise until task failure. Subjects performed isometric knee extension exercise on a dynamometer, allowing assessment of peripheral fatigue at task failure. After 8 min of recovery, subjects completed a second exercise bout, also to task failure. We hypothesized that evoked peak force would be lower at task failure in the second trial compared to the first one, showing that the first exercise bout did not terminate because a critical peripheral fatigue threshold had been reached.

## Materials and methods

### Subjects

Ten sport students (five men, five women, mean ± SD age: 24 ± 4 years, body mass: 71 ± 12 kg, height: 176 ± 9 cm) participated in the study. Subjects were trained in both endurance and strength exercises and classified as performance level 3 or 4 (De Pauw et al., 2013; Decroix et al., 2016). None of the subjects had any leg injury or knee pain. Subjects were instructed to refrain from high-intensity exercise on the day prior to testing and to refrain from alcohol during the last 24 h before testing. Subjects were also instructed to eat a light meal 2–4 h before arrival to the laboratory. The study was approved by the Regional Ethics Committee in Norway (2011/1634), and the experiments were performed according to the latest (2013) revision of the Declaration of Helsinki. The subjects gave their written informed consent to participate in the study. Subjects were given a full explanation of the details and rationale of the study and were informed that they were free to withdraw at any time. The possibility that electrical stimulation might cause discomfort was fully explained as was the nature of the risks involved.

### Experimental protocol

Each subject visited the laboratory on two occasions. During the first visit, the subjects were familiarized with the procedures that would be used for assessment of neuromuscular function consisting of electrical stimulation and isometric MVC. In addition, the subjects were familiarized with the experimental trial involving intermittent isometric contractions at 60% of MVC force until task failure with knee extension on the KinCom dynamometer (Kinematic Communicator, Chattecx Corp., Chattanooga, TN). Three to five days after the familiarization visit, subjects visited the laboratory for the experimental trials.

#### Trials to task failure

**Figure 1:**
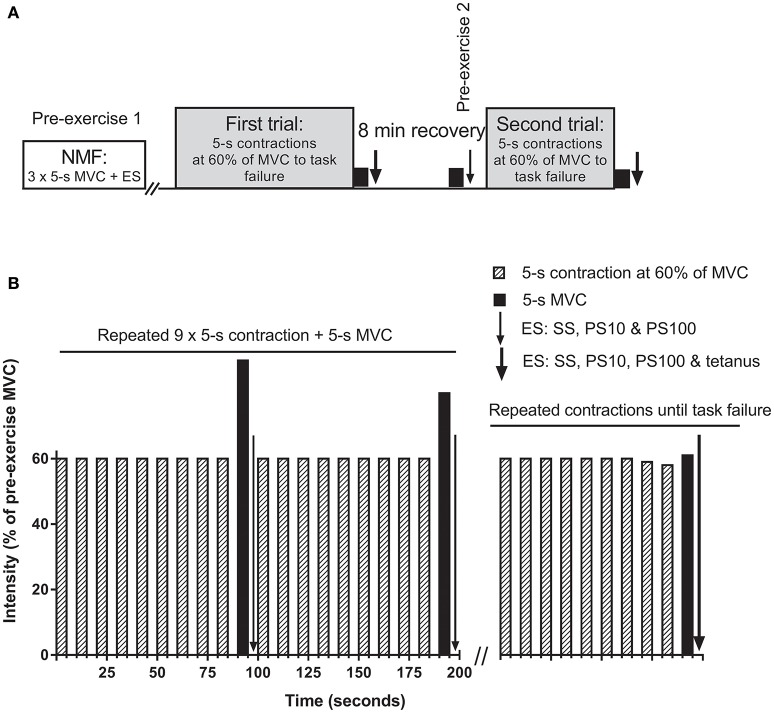
**Overview of the protocol (A)** and detailed description of the trials **(B)**. **(A)** first and second trials were separated by a break of 8 min including the neuromuscular function measurements (NMF). NMF, i.e., a maximal voluntary contraction (MVC) followed within 2 s by electrical stimulation (ES), was assessed three times prior to the first trial (pre-exercise 1), after each set during the trials, at task failure, as well as 1 min before the second trial (pre-exercise 2). **(B)** trials consisted of consecutive sets of 10 × 5-s isometric contractions followed by 5-s rest between contractions. The first nine contractions were performed at a target force at 60% of pre-exercise MVC, while the 10th contraction in each set was a MVC. ES was applied in the 5-s break before the next set of contractions began. Sets of contractions were repeated until task failure. SS, single stimulus; PS10, paired stimuli at 10 Hz; PS100, paired stimuli at 100 Hz; tetanus, 50 stimuli at 100 Hz.

Subjects performed two isometric knee extension trials with the right leg to task failure (Figure 1A), separated by 8 min. One-leg constant load knee extension exercise has been used to investigate the critical peripheral fatigue threshold previously (Amann et al., 2013), but with measurement of peripheral fatigue 2 min after task failure. In the present study, peripheral fatigue assessments began within 2 s following completion of the MVC (i.e., within 7 s post task failure), since we have shown that peripheral fatigue recovers substantially within 1 min after exercise cessation (Froyd et al., 2013), and it is not known if recovery of peripheral fatigue is the same after different exercise trials. During the trials, subjects performed consecutive sets of 10 × 5-s isometric contractions followed by 5-s rest between contractions (Figure 1B). The first nine contractions were performed at a target force at 60% of pre-exercise MVC, while the 10th contraction in each set was a MVC. Electrical stimulation to assess neuromuscular function was applied after each MVCs in each set. A target line on a 24-inch widescreen monitor, positioned in front of the subject, was used for visual feedback of the force recordings during both trials. Task failure occurred when the subject could not maintain the required force for at least 4 s for two consecutive contractions, with subjects being informed each time they failed to achieve the required force output. The experimenter made the decision when task failure had occurred. Following the second missed contraction, subjects were instructed to produce a final 5-s MVC, followed (2 s) by the electrical stimulation protocol described below.

#### Settings and warm-up

On arrival at the laboratory, subjects were secured to the dynamometer by chest and hip strapping to avoid excessive lateral and frontal plane movements. The seating was adjusted for each subject, with the right knee femoral epicondyle aligned with the axis of the dynamometer's rotation arm. The right lower leg was attached to the lever arm just above the lateral malleolus. The left leg was not active at any time and was secured to the dynamometer by strapping around the upper leg. The seat's backrest was reclined 10 degrees, and the dynamometer's rotation arm was kept at 90 degrees. Hip and knee angle was approximately 110 and 80 degrees, respectively. Subjects kept their hands crossed in front of their upper body and in the same position during all experiments.

Warm-up consisted of 5-s isometric contractions followed by 5-s rest. The intensity was 25% of MVC force for five contractions, 50% of MVC force for five contractions, and 75% of MVC force for two contractions. MVC force from the familiarization visit was used to determine warm-up intensity. The rest period between each set was 30 s.

#### Neuromuscular function assessment

Neuromuscular function assessment consisted of a 5-s MVC followed by a sequence of electrical stimuli. For the MVC, the subjects were instructed to produce maximal force for 5 s whilst they received strong verbal encouragement. Femoral nerve electrical stimulation on relaxed muscles consisted of single stimulus (SS), paired stimuli at 10 Hz (PS10), and paired stimuli at 100 Hz (PS100), and assessment started within 2 s after a MVC. The interval between the stimulation techniques was 1.5 s. Hence neuromuscular function assessment duration excluding MVC was approximately 3.5 s. In addition, PS100 was followed by tetanus (50 stimuli at 100 Hz = 0.5 s) once prior to the first trial and once at task failure of both trials. Thus, electrical stimulation lasted from second 2–7 after the MVC at task failure.

Pre-exercise neuromuscular function (Figure 1A) assessment started 2 min after the warm up. Three isometric MVCs, each lasting 5 s were performed with a 2 min break between MVCs and followed by electrical stimulation. Neuromuscular function was also assessed after each set during the trials, at task failure, and 1 min prior to the start of the second trial. Power Lab (ADInstruments Pty Ltd, Bella Vista NSW, Australia) was used to trigger the electrical stimulation.

### Data collection

#### Electrical stimulation

A high voltage (maximal voltage 400 V) constant current stimulator (DS7AH, Digitimer, Hertfordshire, UK) was used to deliver square-wave stimuli of 1 ms duration. The femoral nerve was stimulated percutaneously via a 10 mm diameter self-adhesive cathode electrode (Skintact, Austria) pressed manually by the investigator onto the skin at the femoral triangle. The anode, a 130 × 80 mm self-adhesive electrode (Cefar-Compex Scandinavia AB, Sweden), was applied to the gluteal fold. The optimal stimulation intensity for one single stimulus was determined by increasing the current gradually from 10 mA until a plateau in force was reached. The current was then increased by a further 30% (current range: 35–60 mA) to ensure supramaximal stimulation. The intensity was kept constant for the same subject for all types of electrical stimulation. The subjects were instructed to relax fully whilst the electrical stimulation was applied.

#### EMG recordings

EMG signals from the *vastus lateralis* and *vastus medialis* of the right leg were recorded via surface electrodes (DE-2.1 single differential surface sensors, distance between muscle site contacts = 10 mm; Delsys Inc, Boston, MA). SENIAM (Merletti and Hermens, 2000) recommendations were used for the placement of the sensors on the skin. The skin was shaved and wiped with isopropyl alcohol before the sensors were applied. The reference electrode was applied to the patella. EMG signals were sampled at 2000 Hz and amplified (gain = 1000) using Bagnoli-8 (Delsys Inc). EMG signals were transferred together with simultaneous force and electrical stimulation recordings into Power Lab (ADInstruments) and filtered using a band pass filter with a bandwidth at 15–500 Hz in Lab Chart Pro software (ADInstruments).

#### RPE

Perceived exertion (also known as perception of effort) defined as “the conscious sensation of how hard, heavy, and strenuous exercise is” (Pageaux, 2016), was assessed after every 8th contractions in each set for the trials using the ratings of perceived exertion (RPE) scale (Borg, 1974). Standardized instructions for the scale were given to subjects before the warm-up. Subjects were asked to rate how hard they were driving their leg during the exercise, but not to include an expression of pain in their legs.

### Experimental variables and data analysis

#### Force data

Mean of the three successful MVCs prior to the first trial of exercise was taken as the pre-exercise MVC. Pre-exercise MVC force was used for calculation of the target force at 60% of MVC in both trials. MVC force was calculated as the highest average force sustained for 1 s. Force was also calculated for the first nine contractions of each set by averaging the force during the middle 4 s of the 5 s contractions. The force responses to electrical stimulation are reported as evoked peak force. The mean value in evoked peak force after the three MVCs was therefore used as the pre-exercise value. A reduction in evoked peak force, highlighting peripheral fatigue development, is due to factors distal to the site of stimulation, that is, at the neuromuscular junction or within the muscle. PS10/PS100 (evoked peak force for PS10/PS100) was calculated as an index of low-frequency fatigue (Verges et al., 2009).

#### EMG

The root mean square (RMS) of the EMG data of *vastus lateralis* and *vastus medialis* was calculated for 1 s around peak force for MVC, i.e., 500 ms before and after peak force, and for the middle 4 s of the first nine contractions of each set. M-wave peak-to-peak amplitude in response to SS was also assessed. RMS during voluntary contractions was normalized to RMS of pre-exercise MVC. In addition RMS during voluntary contractions was divided by the M-wave peak to peak amplitude of the following SS response to estimate neuromuscular activation (Millet et al., 2011). To limit the number of MVCs at task failure, voluntary activation was not assessed to calculate the extent of central fatigue.

### Statistical analyses

After checking for the normality of data distribution using the Shaprio-Wilk's test, one-way repeated-measures ANOVAs with Bonferroni *post hoc* corrections were used to detect differences over time (pre-exercise 1, task failure first trial, pre-exercise 2, and task failure second trial; Table 1). Where the assumption of sphericity (Mauchy's test) was violated, the Greenhouse-Geisser Epsilon correction was applied to the degrees of freedom. A paired samples student's *t*-test was used for the following pairwise comparisons for differences at task failure between the two trials for neuromuscular function parameters expressed as a percent of baseline values (Table 1); the slope of within-participants RPE values between trials; and set force during the last set between the two trials. Differences in RPE values after the first set of contractions and at task failure between trials was analyzed using a one-way repeated-measures ANOVA with Bonferroni *post hoc*. The statistical significance was defined at *P* < 0.05. Effect sizes are given as Partial Eta Squared for the ANOVA and Cohen's dz for the paired *t*-tests. All analyses were performed using SPSS version 23 (SPSS, Inc., Chicago, IL), except for paired samples student's *t*-test and Cohen's dz (Microsoft Excel 2013, Microsoft Corporation, WA). The results are presented as mean ± SD.

**Table 1:** **Effects of knee extensors intermittent isometric time to task failure on knee extensors neuromuscular function**.

	**Pre-exercise 1**	**Task failure first trial**	**Pre-exercise 2**	**Task failure second trial**	**F**	***P***-**value**	**Effect size**
MVC (N)	547 ± 123	346 ± 72 ^+++^	410 ± 102 ^+++^	336 ± 62 ^+++^, ^‡‡^	(1.55, 13.97) = 56.75	< 0.001	$\eta_{\text{p}}^{2}$ =.863
Δ%		−36 ± 8		−38 ± 8		0.216	Dz = 0.42
SS (N)	152 ± 48	70 ± 17 ^+++^	93 ± 28 ^+++^	62 ± 16 ^+++^, ^*^, ^‡‡^	(1.15, 10.39) = 43.17	< 0.001	$\eta_{\text{p}}^{2}$ =.827
Δ%		−52 ± 11		−57 ± 10^**^		0.005	Dz = 1.15
PS10 (N)	242 ± 72	107 ± 27 ^+++^	133 ± 43 ^+++^	92 ± 28 ^+++^, ^**^, ^‡‡^	(1.19, 10.75) = 51.19	< 0.001	$\eta_{\text{p}}^{2}$ =.850
Δ%		−54 ± 11		−60 ± 10^***^		< 0.001	Dz = 1.65
PS100 (N)	231 ± 62	137 ± 29 ^+++^	166 ± 42 ^+++^	123 ± 30 ^+++^, ^***^, ^‡‡‡^	(1.10, 9.91) = 47.20	< 0.001	$\eta_{\text{p}}^{2}$ =.840
Δ%		−39 ± 10		−45 ± 10^***^		< 0.001	Dz = 2.02
PS10/PS100	1.04 ± 0.07	0.78 ± 0.08 ^+++^	0.79 ± 0.11 ^+++^	0.75 ± 0.09 ^+++^	(1.05, 9.46) = 32.02	< 0.001	$\eta_{\text{p}}^{2}$ =.781
Δ%		−25 ± 6		−28 ± 7		0.072	Dz = 0.65
Tetanus (N)	471 ± 141	356 ± 102 ^++^	NA	314 ± 101 ^+++^, ^***^	(3, 27) = 62.76	< 0.001	$\eta_{\text{p}}^{2}$ =.875
Δ%		−23 ± 10		−32 ± 11^***^		< 0.001	Dz = 2.46
MVC RMS·M^−1^ *VL*	0.057 ±.012	0.063 ± 0.023	0.054 ± 0.014	0.070 ± 0.020	(1.54, 13.84) = 4.18	0.046	$\eta_{\text{p}}^{2}$ =.317
Δ%		11 ± 25		25 ± 28		0.098	Dz = 0.58
MVC RMS·M^−1^ *VM*	0.072 ±.015	0.079 ± 0.024	0.067 ± 0.017 ^+^	0.085 ± 0.023 ^‡^	(3, 27) = 7.29	0.001	$\eta_{\text{p}}^{2}$ =.448
Δ%		9 ± 17		18 ± 16		0.224	Dz = 0.41
PPA *VL* (mV)	3.75 ± 1.03	3.62 ± 1.01	3.25 ± 0.90 ^+^	3.45 ± 0.82	(3, 27) = 3.51	0.002	$\eta_{\text{p}}^{2}$ =.421
Δ%		−3 ± 10		−7 ± 13		0.249	Dz = 0.39
PPA *VM* (mV)	3.80 ± 0.61	3.55 ± 0.81	3.18 ± 0.59 ^+^	3.47 ± 0.92	(1.53, 13.73) = 6.44	0.015	$\eta_{\text{p}}^{2}$ =.417
Δ%		−7 ± 14		−9 ± 17		0.340	Dz = 0.32

*Values are expressed in absolute units and as a percentage change from pre-exercise 1 (Δ%). Values are expressed as means ± SD (n = 10). MVC, maximal voluntary contraction; SS, single stimulus; PS10, paired stimuli at 10 Hz; PS100, paired stimuli at 100 Hz; Tetanus, 50 stimuli at 100 Hz; RMS, root mean square; M, M-wave; VL, vastus lateralis; VM, vastus medialis; PPA, peak to peak amplitude of the M-wave; NA, not assessed. Significant difference compared with pre-exercise 1*;

+ *P < 0.05*,

+++ *P < 0.001*;

*significant difference between task failure of the first trial and task failure of the second trial*:

* *P < 0.05*,

** *P < 0.01*,

*** *P < 0.001*;

*significant difference between pre-exercise 2 and task failure of the second trial: ^†^ P < 0.05*,

‡‡ *P < 0.01*,

‡‡‡ *P < 0.001*.

## Results

Target force for each set of contractions was predetermined by the protocol, however it was slightly (~1%) higher during the last set of contractions for the first compared with the second trial (311 ± 49 vs. 308 ± 47 N respectively, *t*(9) = 2.38, *P* = 0.041, *dz* = 0.97). As expected, time to task failure was longer (*P* < 0.001) during the first compared with the second trial (12.84 ± 5.60 vs. 5.74 ± 1.77 min, *t*(9) = 5.45, *P* < 0.001, *dz* = 1.72), indicating a 55 ± 16% reduction in time to task failure between the first and second trials.

**Figure 2:**
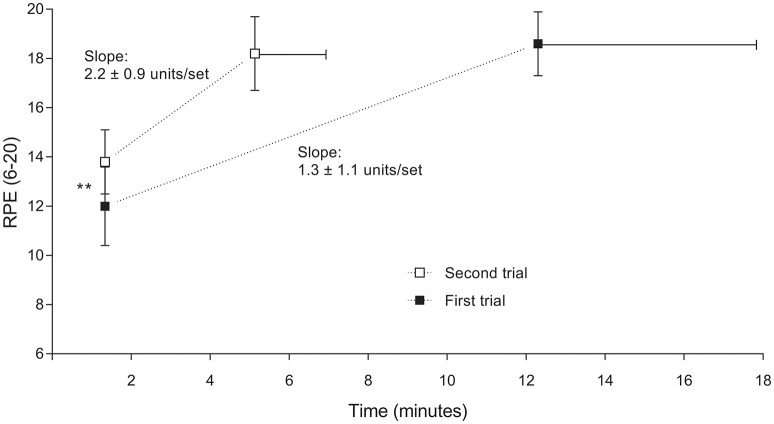
**Changes of ratings of perceived exertion (RPE) during the first and second trial, from the first to the last completed contraction set**. Data are shown as mean ± SD, *n* = 10. Significant difference between first and second trial: ^**^ *P* < 0.01. RPE during the last set was not different (*P* = 0.517) between trials.

RPE was lower at the end of the first set for the first compared with the second trial (12.0 ± 1.6 vs. 13.8 ± 1.3, *P* = 0.020; Figure 2). During the last set of contraction however RPE was not different between trials (18.6 ± 1.3 vs. 18.2 ± 1.5 for first and second trial respectively, *P* = 0.517). A comparison of the within-subjects RPE slopes between conditions indicated that rate of increase in RPE was higher for the second compared with the first trial (2.2 ± 0.9 vs. 1.3 ± 1.1 units/set respectively, *t*(9) = 6.08, *P* < 0.001, *dz* = 1.91).

### Neuromuscular fatigue

Neuromuscular function responses as absolute values prior to the first trial, 1 min prior to the second trial, at task failure in the first and second trials, and in addition at task failure in the first and second trials expressed in percentage of pre-exercise of the first trial, are presented in Table 1. MVC force was significantly lower at task failure for both trials compared with pre-exercise 1 (both *P* < 0.001), but no differences were observed between the first and the second trial at task failure (*P* = 1.00). Evoked peak force for SS, PS10, PS100, and tetanus were significantly lower at task failure for both trials compared with pre-exercise 1 (all *P* < 0.001). Importantly, these three indices of peripheral fatigue were reduced more at task failure in the second compared with the first trial (*P* = 0.014 for SS, *P* = 0.002 for PS10, and *P* < 0.001 for PS100 and for tetanus). PS10/PS100 was significantly lower at task failure for both trials compared with pre-exercise 1 (both *P* < 0.001), but no differences were found at task failure between trials (*P* = 0.464). M-wave amplitude was unchanged at task failure for both trials compared to pre-exercise 1 for the *vastus lateralis* (*P* = 1.00 and *P* = 0.589 for first and second trial respectively) and *vastus medialis* (*P* = 1.00 and *P* = 0.853 for first and second trial respectively). RMS·M^−1^ during MVC was not significantly decreased at task failure in any of the trials compared to pre exercise 1 for both *vastus lateralis* (*P* = 1.00 and *P* = 0.182 for first and second trial respectively) and *vastus medialis* (*P* = 0.872 and *P* = 0.079 for first and second trial respectively).

**Figure 3:**
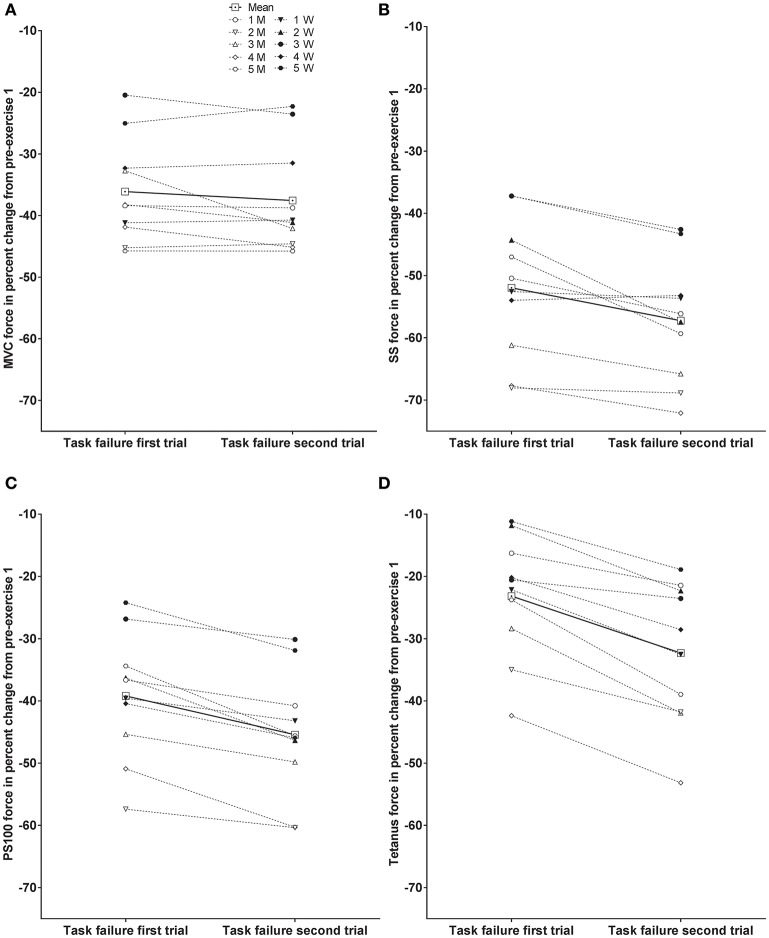
**Individual responses at task failure of the first and second trial as percent change from pre-exercise prior to the first trial; maximal voluntary contraction (MVC) force (A)**, evoked peak force for single stimulus (SS) **(B)**, evoked peak force for paired stimuli at 100 Hz (PS100) **(C)**, and evoked peak force for 50 stimuli at 100 Hz (tetanus) **(D)**. Male subjects (M) are indicated with open symbols, and female subjects (W) are indicated with closed symbols. Significant differences are presented in Table 1.

Individual responses to MVC, SS, PS100, and tetanus force at task failure of the first and second trial as percent change from pre-exercise prior to the first trial are presented in Figure 3. Significant differences between trials are presented in Table 1.

## Discussion

The present study aimed to test the validity of the critical peripheral fatigue threshold model during isometric intermittent contractions of the knee extensors continued until task failure. The most important finding of this study was that evoked peak forces (including tetanus, see below) were reduced to a greater extent at task failure after the second trial compared with the first trial, indicating that task failure in the first trial did not occur in order to ensure that a critical peripheral fatigue threshold was not exceeded during that bout of exercise.

### Peripheral fatigue at task failure

As expected from other studies (Amann and Dempsey, 2008; Neyroud et al., 2012; Amann et al., 2013; Hureau et al., 2014; Johnson et al., 2015), residual neuromuscular fatigue from the first trial contributed to reduced time to task failure during the second trial. However, while no differences in MVC force were found between trials at task failure, in accordance with Neyroud et al. (2012), all electrical stimulation methods including tetanus revealed higher levels of peripheral fatigue at task failure in the second trial.

Two studies have investigated the effect of pre-fatiguing concentric exercise until task failure on the critical peripheral fatigue threshold, and both studies reported differences in peripheral fatigue between trials (Amann et al., 2013; Johnson et al., 2015). In the study of Johnson et al. (2015), subjects cycled on a cycle ergometer to task failure at 85% of peak power with and without pre-fatiguing exercise of the arms. The level of peripheral fatigue was less and the exercise duration was shorter when the arm muscles were pre-fatigued by 8 × 1-min of arm-cranking at a fixed work rate. The finding of Johnson et al. (2015) are in line with another study (Amann et al., 2013), both of which indicate that pre-exercise might prevent the attainment of this so-called critical level of peripheral fatigue. In their study, Amann et al. (2013) compared fatigue induced by concentric single leg knee extension exercise with the same exercise task performed by the same leg following an exhaustive exercise bout with the other leg (Amann et al., 2013). Lower levels of peripheral fatigue were also reported after maximal intermittent dynamic leg extensions (Christian et al., 2014) or constant load cycling (Amann et al., 2007) in hypoxia versus normoxia and after longer than shorter knee extension time trials (Froyd et al., 2016).

Yet, none of these studies absolutely refutes the existence of a critical peripheral fatigue threshold since the experimental conditions differed. To our knowledge, only the present study and that of Neyroud et al. (2012) have compared the level of peripheral fatigue at task failure following a pre-fatiguing isometric trial of the same muscle group. In the latter study (Neyroud et al., 2012), the subjects performed consecutive trials of sustained isometric exercise at 20% of MVC force, interspersed with electrical muscle stimulation. MVC force at task failure was similar between trials and the level of peripheral fatigue at task failure was greater after the second trial. As in the present study, those authors concluded that task failure may not be associated with a critical threshold of peripheral fatigue. Evoked peak forces for SS and PS100 immediately at task failure were reduced equally or slightly more in the present study compared to the data of Neyroud et al. (2012).

In the present study, not only do we confirm these results for voluntary contractions only but we also show that the less frequently used stimulation method of tetanic stimulation (Place et al., 2010) also detected different evoked peak forces at task failure following the two trials. The advantage of tetanic stimulation is that the force response is less affected by potentiation than is the case with single or paired stimuli (Baudry et al., 2008). The measured reduction in evoked peak force was different for the different methods of electrical stimulation (SS > PS100 > tetanus) as previously shown for concentric knee extension exercise, highlighting the importance of the stimulation method used for the quantification of the absolute extent of peripheral fatigue (Froyd et al., 2013, 2016). Nonetheless, the finding of increased peripheral fatigue after the second bout was consistent for all methods of electrical stimulation. More recently, a critical peripheral fatigue threshold has also been questioned by considering individual vs. pooled data (Neyroud et al., 2016).

### Validity of the critical peripheral fatigue threshold

Group III and IV muscle afferents provide inhibitory feedback from locomotor muscles to the central nervous system, presumably influencing the regulation of central motor drive during fatiguing exercise (Taylor and Gandevia, 2008; Amann, 2012). According to this model, humans may not ever exceed a specific level of peripheral fatigue (Amann et al., 2013). As a result, when approaching the critical peripheral fatigue threshold, group III, and IV muscle afferents should begin to inhibit muscle activation and thus cause task failure during constant load exercise (Amann et al., 2011).

Studies have reported similar levels of peripheral fatigue between constant-load cycling trials at 81–83% of peak power in normoxia vs. hypoxia (Amann et al., 2006, 2007), and between constant-load cycling trials at 83% of peak power, a 5 km cycling time-trial, and a 5 km cycling time-trial after pre-fatiguing constant-load cycling to task failure (Amann and Dempsey, 2008). In addition, further support for a critical peripheral fatigue threshold was that more peripheral fatigue was reached following selective blockade of sensory afferents with intrathecal fentanyl injection compared to saline (Amann et al., 2009, 2011; Blain et al., 2016). This suggests that group III and IV afferents might play a critical role in the prevention of dangerous levels of peripheral fatigue. However, despite reaching higher levels of peripheral fatigue, performance was not improved with inhibition of group III and IV muscle afferents with intrathecal fentanyl injection.

Interestingly, in several studies supporting the critical peripheral fatigue threshold, voluntary activation was not reduced after any of the constant load or self-paced endurance trials (Amann et al., 2007, 2011, 2013; Amann and Dempsey, 2008). It is possible that voluntary activation had recovered when measured a few minutes after end of exercise (see below). Even though there was no reduction in voluntary activation measured at task failure with the interpolated twitch technique, the authors (Amann et al., 2013) nevertheless concluded that peripheral fatigue and inhibitory feedback from group III and IV muscle afferents limited the endurance performance by restricting central motor drive to the working muscles. Since a decline in voluntary activation after exercise is indicative of a reduction in central motor drive to the muscles, available data does therefore not indicate that group III and IV muscle afferents inhibited central motor drive. However, a recent study suggests that decreased voluntary activation can be explained by inhibition of type III and IV afferents (Sidhu et al., 2017). Different levels of peripheral fatigue between trials (Neyroud et al., 2012; Amann et al., 2013; Johnson et al., 2015) without differences in voluntary activation are also incompatible with the critical peripheral fatigue threshold model of exercise regulation.

According to this model, endurance exercise performance is limited by a reduced central motor drive and hence force production at exercise termination (Amann et al., 2013). In contrast others argue that performance during endurance exercise does not terminate as a result of peripheral fatigue in the exercising muscles but is due rather to changes in the central nervous system (Marcora and Staiano, 2010; Neyroud et al., 2012, 2016; Morales-Alamo et al., 2015; Froyd et al., 2016). The basis for this conclusion was the finding that subjects were able to increase force production shortly after task failure and before there was any recovery in peripheral fatigue (Marcora and Staiano, 2010; Morales-Alamo et al., 2015). This has led other authors to propose alternative models in which RPE is not—as in the psychobiological model of endurance performance (Marcora et al., 2008; Pageaux, 2014)—or only partly—as in the flush model (Millet, 2011)—explained by the role of feedback from afferent fibers. In the present study and in others (Neyroud et al., 2012; Amann et al., 2013; Johnson et al., 2015), RPE was similar at the end of the trials despite the finding that RPE was higher in the first part of those trials when subjects were pre-fatigued by bouts of prior exercise. Those findings in addition to the differences in RPE slope between trials in the present study, also provide support for the psychobiological model of endurance performance (Marcora et al., 2008; Pageaux, 2014), the flush model (Millet, 2011), and the central governor model (Noakes, 2012).

### Methodological considerations and limitations

While in the present study evoked peak force was assessed following isometric intermittent contractions of the knee extensors to task failure, the critical peripheral fatigue threshold model originated from studies using cycling as the exercise modality (Amann et al., 2006; Amann and Dempsey, 2008). An important limitation in cycling studies is that neuromuscular function is normally assessed several minutes after exercise cessation. In cycling studies investigating the critical peripheral fatigue threshold (Amann et al., 2006, 2009; Amann and Dempsey, 2008; Johnson et al., 2015), evoked peak force was first measured with SS 2–4 min after cycling exercise cessation, at which time values were reduced by 32–38%. These percentage decreases in evoked peak force are very similar to those measured in the present study 7 min after the first trial (pre exercise trial 2, Table 1) and in our previous study, 4 and 8 min after exercise cessation (Froyd et al., 2013). It is probable that in studies in which peripheral fatigue is first assessed, at the earliest, even 2 min after exercise cessation, the absolute level of peripheral fatigue at exercise cessation is underestimated since peripheral fatigue recovers substantially within the first 1–2 min after exercise cessation (Froyd et al., 2013, 2014). Whether or not this findings contributed to the development of the critical threshold model is not known.

Subjects knew beforehand that the experiment comprised of two trials. Even though participants were strongly encouraged to exercise to their maximal capacity in both trials, it is possible that participants might have terminated the first trial in a “submaximal” state of fatigue but were motivated to exercise to higher levels of fatigue in the second trial since they knew that this was the final trial (Halperin et al., 2014). Although this could lead to biased results, this is a common feature of studies involving consecutive trials of exercise (Neyroud et al., 2012; Amann et al., 2013; Johnson et al., 2015). However, RMS·M^−1^, an index of central motor drive, during the last set and during the MVC at task failure were not different between the first and second trial, indicating that participants were not holding back at the end of the first compared to the second trial. In addition RPE values were the same at exercise termination in both trials suggesting equivalent effort.

The experimenter decided that task failure occurred when the developed force dropped below the target force. The experimenter was not blind to the aim of the study and might have allowed the subject to continue working below target force; this would have increased the development of peripheral fatigue. However, during the last set, force production was 1% lower during the second than first trial and RPE was not different between trials, indicating that differences in evoked peak force between trials could not be explained by differences in voluntary force production at target force.

## Conclusions

It has been proposed that task failure during exhaustive endurance exercise is constrained by group III and IV inhibitory feedback from the exercising muscles specifically to ensure that the level of peripheral fatigue is always maintained below some critical threshold. In contrast, in this study we established that task failure occurred at different levels of evoked forces, including tetanus, during consecutive trials of similar target force. This indicates that task failure in the first trial did not occur in order to ensure that a critical peripheral fatigue threshold was not exceeded during that exercise bout.

## Author contributions

The experiments were performed at Sogn og Fjordane University College, Norway. CF and TN conceptualized and designed the study. CF collected the data; CF analyzed the data, while all authors interpreted the data. CF drafted the manuscript, while all authors contributed to the manuscript and approved the final version of the manuscript.

## Funding

This research was funded by the University of Cape Town Staff Research Fund, the Medical Research Council of South Africa, Discovery Health and the National Research Foundation.

### Conflict of interest statement

The authors declare that the research was conducted in the absence of any commercial or financial relationships that could be construed as a potential conflict of interest.

## Abbreviations

EMG: electromyography
MVC: maximal voluntary contraction
PS10: paired stimuli at 10 Hz
PS100: paired stimuli at 100 Hz
PS10/PS100: evoked peak force for PS10/PS100
RMS: root mean square
RMS·M^−1^: root mean square/M-wave peak to peak amplitude
RPE: rating of perceived exertion
SS: single stimulus
Tetanus, tetanic stimulation: 50 stimuli at 100 Hz = 0.5 s.
